# The Regulatory Effect of MicroRNA-101-3p on Disc Degeneration by the STC1/VEGF/MAPK Pathway

**DOI:** 10.1155/2021/1073458

**Published:** 2021-10-05

**Authors:** Juehan Wang, Leizhen Huang, Xi Yang, Ce Zhu, Yong Huang, Xin He, Kun Shi, Jingcheng Wang, Ganjun Feng, Limin Liu, Yueming Song

**Affiliations:** Department of Orthopedic Surgery and Orthopedic Research Institute, West China Hospital, Sichuan University, No. 37 Guoxue Road, Chengdu, Sichuan, China

## Abstract

*Aims*. Accumulating evidence reported that the microRNA (miRNA) took an important role in intervertebral disc degeneration (IDD). In this study, we revealed a novel miRNA regulatory mechanism in IDD. *Main Methods*. The miRNA microarray analyses of human degenerated and normal disc samples were employed to screen out the target miRNA. In vitro and in vivo experiments were conducted to verify the regulatory effect of miR-101-3p. *Key Findings*. The expression level of miR-101-3p was significantly decreased in the degenerated disc samples which were confirmed by qRT-PCR. Moreover, the miR-101-3p expression level was changed dynamically according to the disc degeneration grade. Upregulation of miR-101-3p expression level inhibited cell apoptosis. Furthermore, stanniocalcin-1 (STC1) was selected to be the target gene of miR-101-3p according to the bioinformatic algorithms. Mechanically, upregulation of miR-101-3p significantly decreased the expression of STC1, vascular endothelial growth factor (VEGF), and MAPK pathway expression levels. Therapeutically, in vivo experiment on IDD rat model illustrated that agomir-101-3p could effectively suspend IDD. *Significance*. Our findings demonstrated that miR-101-3p alleviated IDD process through the STC1/VEGF/MAPK pathway.

## 1. Introduction

As a common disease, low back pain (LBP) is the leading cause of disability among the global disabling disorders, placing a severe economic burden on society. According to a report, a total of about 650 million people worldwide are under the influence of low back pain; in the United States, more than $30 billion medical costs are spent each year because of low back pain [1]. Intervertebral disc degeneration (IDD) is one of the main pathogenic factors of low back pain. The intervertebral disc is the largest avascular structure within the human body, which contains soft gelatinous nucleus pulposus (NP) enclosed by a thick layer of annulus fibrosus (AF). IDD is characterized by the structural alteration due to the rupture or injury of annulus fibrosus and the nucleus pulposus prolapse which causes a series of clinical diseases, such as intervertebral disc herniation, spinal canal stenosis, degenerative spondylolisthesis, and scoliosis. Currently, the treatment of IDD is limited to symptomatic treatment and conservative treatment; no progress has been made to effectively slower or reverse the process of degeneration. Therefore, IDD pathogenesis was posed as a profound issue due to the onerous public medical needs and the patients' pain.

Although the exact molecular pathogenesis of IDD remains unclear, several studies have reported that various abnormally regulated cellular events were associated with the pathogenesis of IDD, such as the apoptosis and autophagy of nucleus pulposus, production of inflammatory cytokines, and angiogenesis [2]. In the previous studies, stanniocalcin-1 (STC1) was found to be involved in angiogenic sprouting by regulating the vascular endothelial growth factor (VEGF) [3, 4]. Meanwhile, the VEGF-mediated angiogenesis was considered to be one of the key factors for IDD [5]. However, the comprehensive molecular mechanism about STC1-mediated IDD, particularly how STC1 mRNA was regulated by upstream genes, had not been fully revealed.

MicroRNA is defined as a kind of noncoding single-stranded RNA molecules with the length of 18~22 nucleotides encoded by endogenous genes, which can specifically target and bind the 3′-untranslated regions (3′-UTR) of mRNA in the posttranscriptional period [6, 7]. The purpose of this research was to investigate the exact functions of miRNAs in STC1-mediated IDD.

As the first step of this study, comprehensive miRNA profiles were obtained based on microarray to identify differentially expressed miRNAs in NP tissues. Among those selected IDD-specific miRNAs, the upregulation of miR-101-3p expression in degenerative NP was particularly remarkable, which also showed direct association with the severity of disc degeneration. Furthermore, according to the results of *in vitro* and *in vivo* analyses, STC1 served as the target gene of miR-101-3p, which significantly reduced the NP apoptosis by targeting the STC1/VEGF pathway. Our findings revealed the molecular mechanism to explain how miR-101-3p inhibited the STC1 expression to relieve the IDD by preventing VEGF-mediated angiogenesis.

## 2. Materials and Methods

### 2.1. Nucleus Pulposus (NP) Samples

From February 2017 to February 2020, 183 patients (55.7 ± 7.2, range: 44–69 years) who had degenerative disc disease and treated with discectomy or interbody fusion surgery were recruited for sample collection to carry out the next-generation sequencing.

Normal NP samples were obtained from burst fracture patients with spinal cord surgery. The NP samples were classed into different groups according to the Pfirrmann classification (Table S3) [8]. Samples with grade 1 to grade 3 (G1-3) were defined as low grade, while samples with grade 4 to grade 5 (G4-5) were defined as high grade. This study protocol was approved by the Review Board of West China Hospital, Sichuan University, and in accordance with the Helsinki Declaration. And the written consents from the patients were obtained.

### 2.2. Cell Isolation and Culture

NP cells were isolated according to the established protocol [9]. In brief, NP tissues were washed twice in phosphate-buffered saline (PBS) with 1% penicillin/streptomycin and then cut into 0.3-0.5 mm pieces. Then, digest the NP tissue pieces in 0.25% trypsin (Thermo Fisher Scientific, Inc., Waltham, MA, USA) for 30 min at 37°C. The NP cells were obtained from NP tissues by digestion with 1 mg/mL type II collagenase (Invitrogen, Carlsbad, CA, USA) for 5 h at 37°C. Mononuclear cells were isolated by passing the tissue through a 200-mesh cell strainer, followed by 80%/40% Percoll gradient centrifugation. The isolated NP cells were cultured with DMEM medium with 10% fetal bovine serum (Gibco, Grand Island, NY), 1% L-glutamine, 100 U/mL penicillin, and 100 *μ*g/mL streptomycin (Invitrogen) at 37°C incubator with 5% CO_2_. The culture medium was replaced every two days, and when the cell density reached 80%, all the cells were continuously cultured in the same medium after passage. No difference in morphology was found between the two generations of cells. Then, the second-generation cells were used for subsequent experiments.

### 2.3. Establishment of the IDD Rat Model and Histological Evaluation

12 Sprague-Dawley rats (254.6 ± 15.7 g) at the age of 3 months were randomly selected from the Animal Center of Sichuan University. According to the literatures and previous protocol [10, 11], the rats were divided into three groups randomly: untreated IDD model (IDD; *n* = 4), IDD group treated by agomiR-101-3p (GenePharma Co., Shanghai, China; IDD+agomiR-101-3p; *n* = 4), and sham (sham; *n* = 4). Eight rats of the IDD group and the IDD+agomiR-101-3p group underwent needle puncturing at the coccygeal vertebrae to establish the IDD model. Briefly, anesthesia was achieved and maintained by isoflurane inhalation before all the surgical procedures, and the operative field was prepared in a sterile fashion. After determining the location of the intervertebral disc between the fifth to sixth coccygeal vertebrae (Co_5-6_) of each rat via X-ray, a 21-gauge needle was inserted into the center of the NP percutaneously through the annulus fibrosus in the Co_5-6_ level, followed by 180° rotation and 5 s hold, which was controlled by locking forceps clamped at 5 mm from the needle. Seven days later, the same tail disc of each rat in the IDD group was injected with 10 *μ*L of saline, while each of the IDD+agomiR-101-3p group was injected with 10 *μ*L of agomiR-101-3p by a 26-gauge needle. All efforts were made to minimize suffering. All rats were maintained under pathogen-free conditions and were housed under standard diurnal light/dark conditions, fed a standard commercial diet, and allowed free access to water. Four weeks after injection, a microcomputed tomography (CT) was taken to assess the degree of disc degeneration. All radiograph images were analyzed using ImageJ software (NIH, Bethesda, MD). Disc height index (DHI) was determined as described previously [12]. Then, the rats were euthanized through air embolism and the discs were harvested for analysis. Histological hematoxylin-eosin (HE) and Safranin-O-fast green staining (SO-FG) methods were performed. Histological results for assessing the disc degeneration were qualified by histological scores based on the method of Masuda et al. [13]. The animal experiments were performed following protocol approved by the Sichuan University Committee on the Use and Care of Animals.

### 2.4. qRT-PCR

We followed the methods of Huang et al. and Chen et al. [14, 15]. Total RNA from NP cells was isolated using TRIzol reagent (Invitrogen, Waltham, MA, USA) and RNeasy Mini Kit (Qiagen, Valencia, CA, USA) following the manufacturer's protocol. Then, RNA was eluted in 50 *μ*L of nuclease-free water and stored at −80°C for further analysis. After RNA concentration determination using the NanoDrop ND-1000 Spectrophotometer (NanoDrop Technologies, Wilmington, DE, USA), RNA was reverse transcribed with PrimeScript™ RT Master Mix (TakaRa, Dalian, China) according to manufacturer's instructions. The sequences of primers are listed in Supplementary Table S2. The 2^−*ΔΔ*Ct^ method was used to calculate fold changes of targeted mRNAs. U6 and *β*-actin served as reference genes.

### 2.5. Western Blotting

The proteins were obtained from NP cells by RIPA lysis and extraction buffer (Thermo Scientific). The protein concentrations were determined by using a BCA Protein Assay Reagent Kit (Pierce Biotechnology, Rockford, IL, USA) according to the manufacturer's protocol. Western blot analyses were performed according to standard protocols. Briefly, the proteins were loaded and separated on 10% sodium dodecyl sulfate-polyacrylamide gels. Then, transfer the gels to polyvinylidene fluoride (PVDF) membranes (Amersham, Buckinghamshire, UK). 5% skim milk was used to block the membranes for 2 h at room temperature, and incubate membranes overnight at 4°C with anti-STC1 antibody (AB_2608564, 1 : 1000, Invitrogen, USA), anti-VEGF antibody (AB_2212682, 1 : 1000, Invitrogen, USA), anti-MMP3 antibody (NB100-91878, 1 : 1000, Novus, USA), anti-Col II antibody (NB600-844, 1 : 1000, Novus, USA), anti-IL-1*β* antibody (AB_468396, 1 : 1000, Invitrogen, USA), and anti-*β*-actin antibody (abs118937, 1 : 3000, Absin, China) in 5% bovine serum albumin (BSA) in TBS-T overnight at 4°C. After washing with TBS-T three times, the membranes were incubated with the secondary antibody for 2 h at room temperature. Protein bands were detected by using the ChemiDoc™ XRS+ System (Bio-Rad Laboratories, Inc., USA) according to the manufacturer's specifications.

### 2.6. Cell Transfection and Efficiency

Before transfection, NP cells were seeded at a density of 2 × 10^4^ cells per well in a 24-well plate and then incubated overnight for cell attachment. The samples were sorted into groups and transfected with either one of Cy3-labeled miR-101-3p mimic, Cy3-labeled miR-101-3p inhibitor, Cy3-labeled mimic control, or Cy3-labeled inhibitor control (GenePharma, Shanghai, China). NP cells were transfected by Lipofectamine 2000 (Invitrogen, Carlsbad, CA, USA) at a concentration of 20 nM per well according to the manufacturer's protocol. For efficacy evaluation, dual-luciferase assays were performed by using the Dual-Glo Luciferase Assay System (Promega, Wisconsin, USA) at 48 h after transfection. The activities of wild- or mutant-type STC1 3′-UTR reporter plasmid were measured. All abovementioned assays were repeated three times to minimize random error.

### 2.7. Immunocytochemistry (ICC)

Immunocytochemistry was performed following standard protocols. In brief, NP cells were collected and seeded in a 24-well plate and then washed three times with PBS, fixed with 4% paraformaldehyde for 20 min, permeabilized with 0.25% Triton X-100 for 5 min, and blocked with 5% bovine serum albumin for 30 min. Subsequently, cells were incubated with anti-collagen type II (ab34712, 1 : 100, Abcam, UK) and anti-MMP3 (ab52915, 1 : 100, Abcam, UK) at 4°C overnight. Cells were washed with PBS twice and incubated with the secondary antibody IgG-Rhodamine (1 : 200 dilution) (Sigma-Aldrich) for 2 h at room temperature. Nuclei were stained with 4,6-diamidino-2-phenylindole (DAPI; Beyotime, China). Fluorescence images were observed under a fluorescent microscope (Zeiss Axioplan microscope, Carl Zeiss Microscopy, Thornwood, NY, USA).

### 2.8. miRNA Microarrays

The total RNA in disc samples qualified by NanoDrop 2000 spectrophotometer (Thermo Scientific, USA) were used to establish the small RNA libraries using TruSeq RNA Sample Prep Kit v2 (Illumina, USA), TruSeq SR Cluster Kit v3-cBot-HS (Illumina, USA), TruSeq SBS Kit v3-HS (50 cycles) (Illumina, USA), and Quant-iT™ PicoGreen® dsDNA Assay Kit (Life, USA) following the manufacturers' protocols. The libraries were paired-end sequenced with HiSeq 2500 (Illumina). The differentially expressed mRNAs were identified using GeneSpring software version 13.0 (Agilent, CA) after expression data of mRNAs were filtered and normalized. The functional enrichment analysis was carried out using the KEGG Orthology-Based Annotation System (KOBASS).

### 2.9. Flow Cytometry

In order to evaluate the apoptosis, flow cytometry by annexin V-fluorescein isothiocyanate (FITC)/propidium iodide (PI) double staining was conducted according to the manufacturer's instructions. NP cells were washed with cold PBS solution twice and then resuspended in 1x binding buffer. NP cells were stained with 5 *μ*L annexin V-FITC and 5 *μ*L PI; the staining cells were detected and sorted by the CytoFLEX flow cytometer (Beckman Coulter, CA, USA). Cells that were negatively stained for both annexin V-FITC and PI were considered normal. Cells that were positively stained with annexin V-FITC and negatively stained for PI were considered apoptotic. Cells that were positively stained for both annexin V-FITC and PI were considered necrotic.

### 2.10. Gene Ontology (GO) and Kyoto Encyclopedia of Genes and Genomes (KEGG) Enrichment Analysis

For functional enrichment analysis, all DEGs (differentially expressed genes) were mapped to terms in the GO databases, and then, significantly enriched GO terms were searched for among the DEGs using *p* < 0.05 as the threshold. GO term analysis was classified into three subgroups, namely, biological process (BP), molecular function (MF), and cellular component (CC). All DEGs were mapped to the KEGG database and searched for significantly enriched KEGG pathways at *p* < 0.05 level.

### 2.11. CCK-8 Assay

Cell proliferation was measured by CCK-8 assay (CCK-8, Dojindo, Japan). Cell suspensions were seeded onto 96-well plates (3 × 10^3^ cells/well) in triplicate and cultured for 0, 24, 48, and 72 hours. Four hours before absorbance measuring, 10 *μ*L of CCK-8 solution was added. The absorbance was measured at 450 nm with a microplate reader after being incubated at 37°C for 2 h.

### 2.12. Statistical Analysis

SPSS 17.0 (SPSS Inc., Chicago, IL) conducted the calculation processes for the statistical tests, and the GraphPad Prism 8 Software (GraphPad Software, San Diego, CA, USA) was used for graphical representation. Student's *t*-test or one-way ANOVA with Tukey's post hoc test was employed for statistical difference.

## 3. Result

### 3.1. Identification of IDD-Associated miRNAs by Microarray

**Figure 1:**
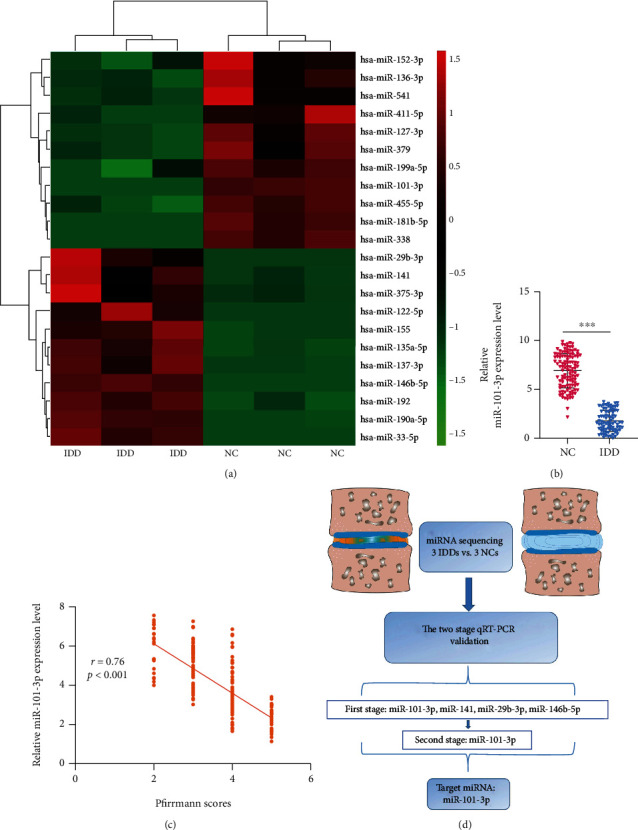
Differentially expressed miRNAs in NP tissues from IDD and normal samples. (a) Heat map depicting 22 differentially expressed miRNAs (fold change > 5 or <0.2, *p* < 0.01). (b) The different expression levels of miR-101-3p analyzed by qRT-PCR assay. (c) The miR-101-3p expression level was changed dynamically and negatively correlated with Pfirrmann scores in IDD (*r* = 0.76, *p* < 0.0001). (d) Schematic diagram showed the selection strategy according to the miRNA microarrays results. ^∗∗∗^*p* < 0.001.

Among the 590 candidate miRNAs selected with sequencing, 101 miRNAs showed abnormal expression in IDD patients compared to the normal control group, which included 56 upregulated and 45 downregulated miRNAs. Only miRNAs with a mean fold change > 5 or <0.2 and a *p* value < 0.01 were selected for further analysis. Accordingly, 22 miRNAs were considered eligible for further investigation; 11 miRNAs were upregulated and downregulated, respectively, in the normal control and IDD NP tissues (Figure 1(a)).

According to the criteria mentioned above, quantitative reverse transcriptase-PCR (qRT-PCR) was carried out to verify the levels of these miRNA expressions in order to further analyze the differential expression. miR-101-3p, miR-141, miR-29b-3p, and miR-146b-5p expression levels were significantly different compared to the other miRNAs. Thereafter, these four miRNAs above were further analyzed by qRT-PCR using an additional independent cohort consisting of 113 IDD samples and 101 controls. Among the four miRNAs, miR-101-3p was found to be significantly downregulated in IDD patients compared with controls and the miR-101-3p differential expression level showed consistent in the first- and second-stage validations (Figure 1(b), Supplementary Table S1). Therefore, miR-101-3p was selected to be analyzed in further experiments. Furthermore, the increased miR-101-3p expression showed a negative correlation to the grade of IDD (*r* = 0.76, *p* < 0.001), as shown in Figure 1(c). These results suggested the possibility that miR-101-3p was deeply involved in the IDD process. The whole selection and validation strategy are shown in Figure 1(d).

### 3.2. The Effect of miR-101-3p Upregulation or Inhibition on NP Cells

**Figure 2:**
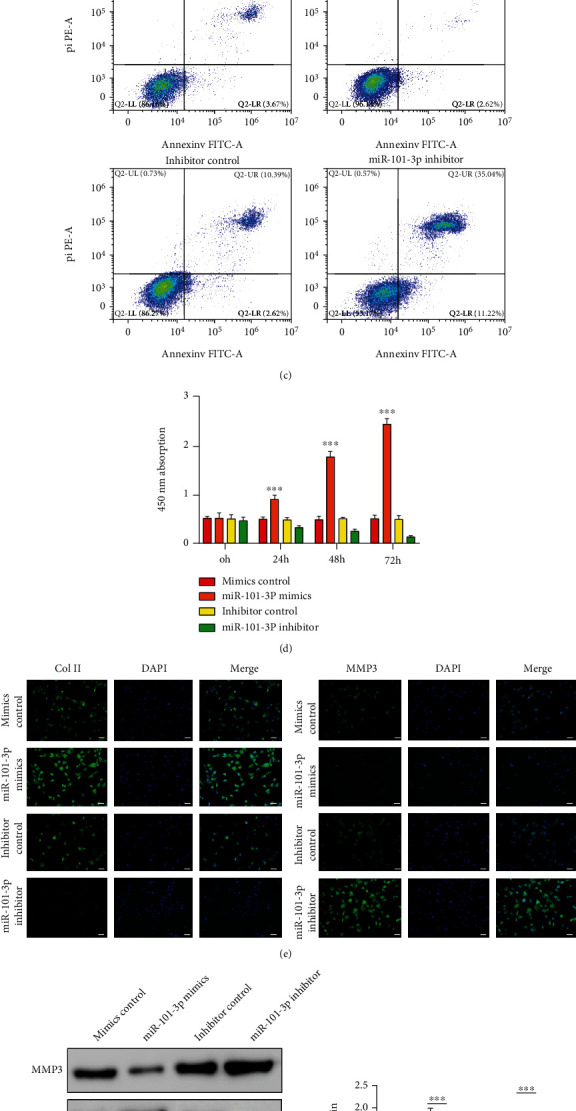
The effect of miR-101-3p upregulation or inhibition on NP cells. (a) The miR-101-3p transfected NP cells confirmed by Cy3, scale bar = 25 *μ*m. (b) The miR-101-3p expression level evaluated by qRT-PCR at 48 h after transfection. *n* = 3 replicates per group. (c) The cell apoptosis was accessed by flow cytometry. (d) Cell viability by CCK-8. (e) The Col II and MMP 3 expression was detected by immunofluorescence. Scale bar = 50 *μ*m. (f) The expression levels of MMP3 and Col II proteins were detected by western blots. Quantitative analysis is shown on the right. Values presented as mean ± SD. ^∗∗∗^*p* < 0.001. NP: nucleus pulposus.

To better understand the functional role of miR-101-3p in the pathogenesis of IDD, miR-101-3p mimics and inhibitors were transfected into the cultured human NP cells. Transfection efficiency was detected by Cy3-labeled miRNA (Figure 2(a)), and then, qRT-PCR was carried out to further confirm the expression of miR-101-3p (Figure 2(b)). At 72 h after the transfection with miR-101-3p mimics and inhibitor, cell apoptosis was significantly inhibited in the group of miR-101-3p mimic transfection while it was enhanced in the miR-101-3p inhibitor group compared to the NC (*p* < 0.001), as shown by Figure 2(c). Meanwhile, the cell viability was markedly enhanced in the group of miR-101-3p mimics according to the CCK-8 assay (Figure 2(d)).

Therefore, in order to further verify the biological effect after miR-101-3p transfection, the expression levels of conventional anabolic/catabolic intervertebral disc markers were measured. As reported, type II collagen (Col II) was the main extracellular matrix component within the disc and the MMP-3 was one of the matrix metalloproteinases which would increase when intervertebral disc degenerated. The expression of COL2 and MMP-3 was evaluated in NP cells which were transfected with miR-101-3p mimics or miR-101-3p inhibitor by immunofluorescence. Upregulation of miR-101-3p significantly increased COL2 levels, whereas inhibition of miR-101-3p decreased them (Figure 2(e)). In contrast, the level of MMP-3 was decreased in the miR-101-3p mimic group and increased in the miR-101-3p inhibitor group (Figure 2(e)). The same results were confirmed by western blots of each corresponding protein (Figure 2(f)). In general, these results above indicated that the upregulation of miR-101-3p promotes NP cell matrix synthesis and proliferation.

### 3.3. Identifying STC1 as the Gene Target for miR-101-3p

**Figure 3:**
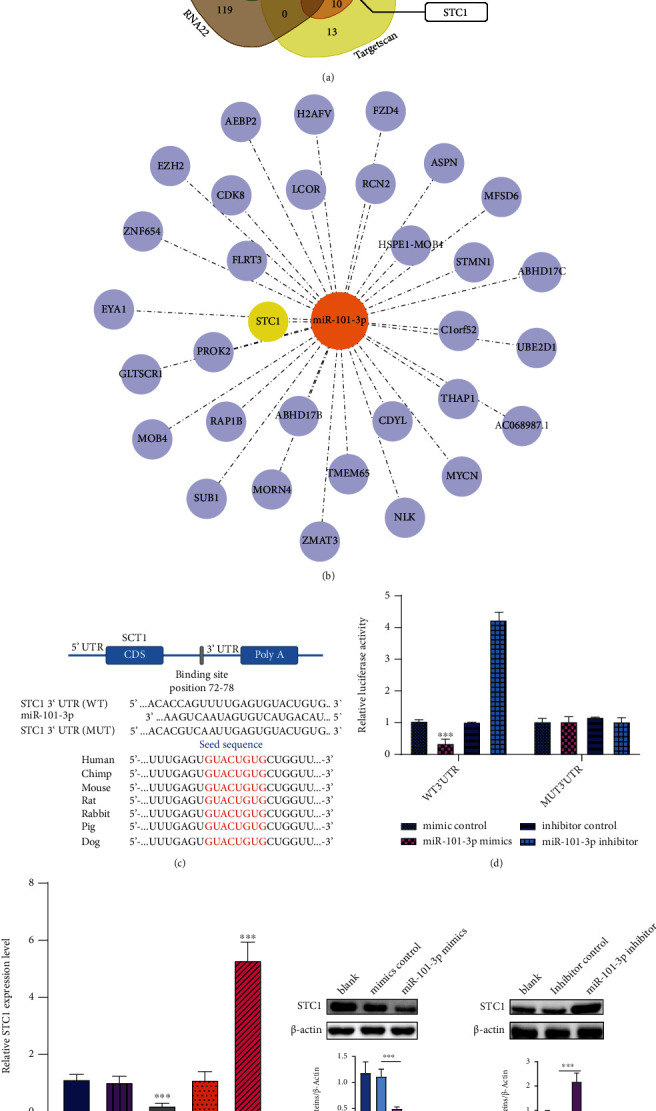
Identification of STC1 as a target of miR-101-3p. (a) Venn diagram showing miR-101-3p computationally predicted to target STC1 by different algorithms. (b) The potential target of miR-101-3p accessed by Cytoscape. (c) Schematic diagram showed that the putative binding site of miR-101-3p within the 3′-UTR of STC1 mRNA demonstrated the high conservation of miR-101-3p between different species. (d) Luciferase activity of wild- or mutant-type STC1 3′-UTR reporter plasmid after transfections in NP cells. (e) The relative STC1 expression level evaluated by qRT-PCR after transfections. (f) The STC1 protein expression accessed by western blots after transfections. Quantitative analysis is shown on the right. Values presented as mean ± SD. ^∗∗∗^*p* < 0.001. NP: nucleus pulposus.

All the predicted target genes were compiled for Venn analysis by TargetScan (https://www.targetscan.org/), miRanda (https://www.microrna.org/), PITA (https://genie.weizmann.ac.il/), RNA22 (https://cm.jefferson.edu/rna22/), and PicTar (https://pictar.mdc-berlin.de/) (Figure 3(a)). The result showed that the sequences of the 3′-UTR of STC1 was highly complementary with miR-101-3p according to the final screening results, which indicates that STC1 might be a putative downstream target gene of miRNA-101-5p. Thereafter, the miRNA–mRNA network was constructed by the Cytoscape software (Figure 3(b)). To verify the correlation between miR-101-3p and STC1, luciferase reporter assays were conducted. The relative luciferase reporter activity of cotransfected STC1 wild type (WT) with miR-101-3p mimics in NP cells was significantly lower than that of which transfected STC1 mutant (MUT) with miR-101-3p mimics (*p* < 0.001), indicating that miR-101-3p mimic could effectively inhibit the luciferase activity of WT plasmid. Furthermore, mutation of the predicted seed sequence of miRNA-101-3p on STC1 3′-UTR salvaged this effect (Figure 3(d)). Then, the STC1 and its corresponding protein expression were also confirmed to be downregulated in the miR-101b-3p mimic group by qRT-PCR and western blots (Figures 3(e) and 3(f)). In general, the aforementioned results validated that STC1 was a direct target of miR-101-3p.

### 3.4. miR-101-3p Regulates IDD by the STC1/VEGF/MAPK Signaling Pathway

**Figure 4:**
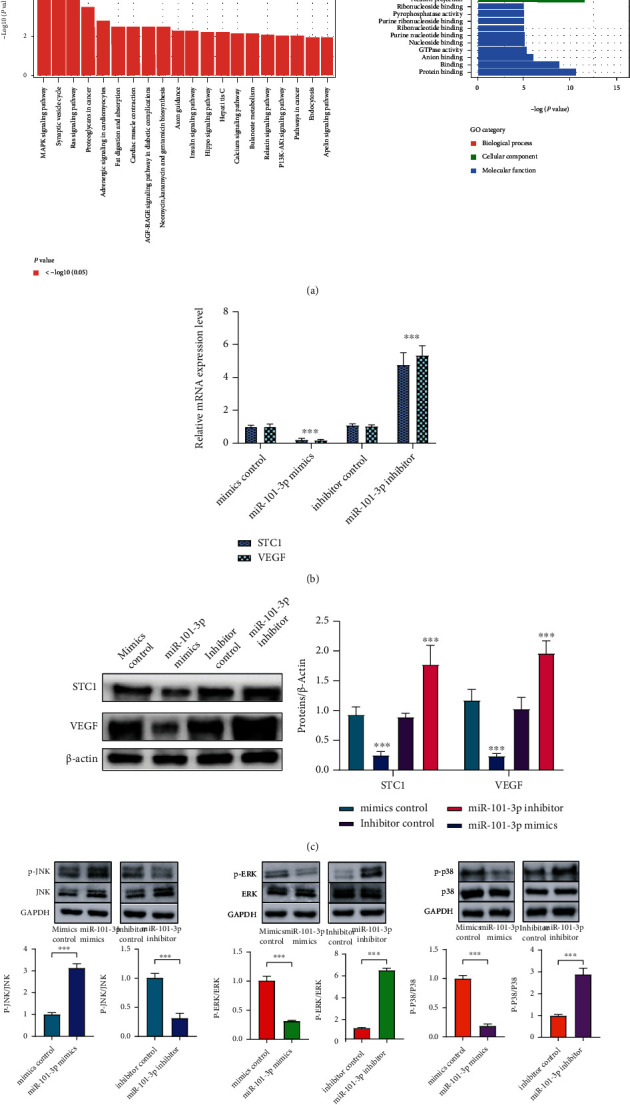
The molecular mechanism of miR-101-3p on the STC1/VEGF/MAPK pathway. (a) KEGG and GO analysis which demonstrated that the MAPK pathway was enriched in IDD. (b) The expression levels of STC1 and VEGF in different transfection groups accessed by qRT-PCR. (c, d) The protein expression levels of STC1, VEGF, p-p38/p-38, p-JNK/JNK, and p-ERK/ERK measured by western blotting. Quantitative analysis is shown alongside. Values presented as mean ± SD. ^∗∗∗^*p* < 0.001.

As shown in Figure 4(a), the MAPK signaling pathway was significantly enriched in Kyoto Encyclopedia of Genes and Genomes pathways. In addition, accumulating evidence indicates that STC1 has regulatory functions on angiogenesis through the VEGF/VEGFR2 pathway [3, 16, 17]. Moreover, the MAPK signaling pathway is one of the conventional downstream pathways of the VEGF/VEGFR2 pathway [18–20]. Since we have revealed that STC1 was a direct target of miR-101-3p above, these findings prompted us to further investigate the potential association between miR-101-3p and the STC1/VEGF/MAPK pathway.

To clarify how miR-101-3p regulated the VEGF/MAPK pathway, the expression of VEGF, p-p38/p-38, p-JNK/JNK, and p-ERK/ERK, which were key factors in the MAPK pathway, in each group, was accessed by qRT-PCR together with western blots. Besides, a qRT-PCR assay was carried out to evaluate the STC1 and VEGF expression level in normal and degenerated disc samples to verify the interaction between STC1 and VEGF. In addition, interaction between proteins was studied using the Search Tool for the Retrieval of Interacting Genes/Proteins (STRING) and its database of known protein interactions (http://string-db.org) to show the interaction between STC1 to VEGF and MAPK (Figure S1).

The expression level of STC1, VEGF, p-ERK, and p-p38 was significantly decreased in the miR-101-3p mimic group while STC1, VEGF, p-ERK, and p-p38 were upregulated in the miR-101-3p inhibitor group (Figures 4(b)–4(d)), which also indicated that miR-101-3p could inhibit the expression of STC1, thereby inhibiting VEGF and downstream MAPK pathway expression.

### 3.5. Upregulation of miRNA-101-3p Reversed the IDD

**Figure 5:**
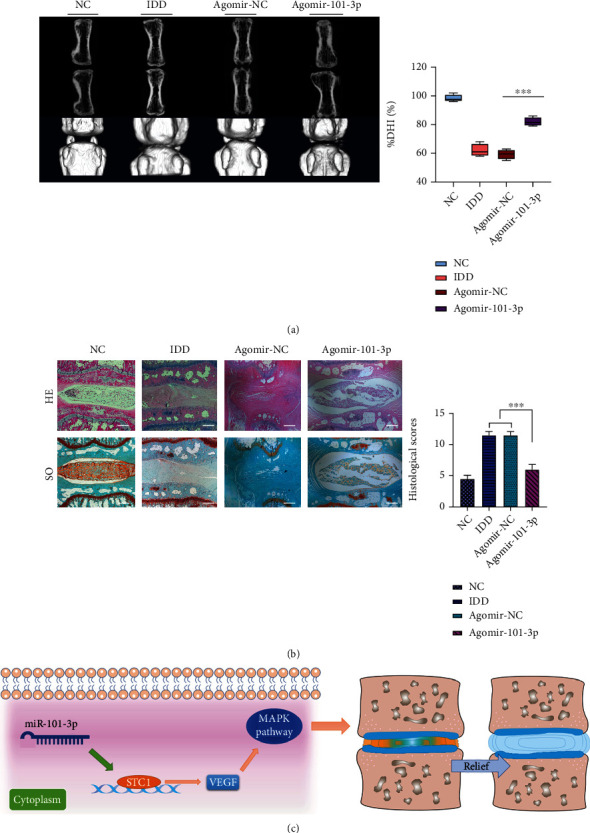
In vivo experiment showed the therapeutic effect of miR-101-3p in IDD rats. (a) The micro-CT radiographic images in each group. Quantitative disc height index (DHI) analysis is shown on the right. (b) HE and Safranin-O-fast green staining of each group's rat disc. Histologic grading scores are shown on the right. (c) Schematic diagram showed miR-101-3p attenuated the degeneration process on the intervertebral disc tissues. Data are shown as mean ± SD of four rats in each group. Sham: sham surgery group; IDD: IDD model group; IDD+agomiR-101-3p: IDD model treated with antagomiR-101-3p. ^∗∗∗^*p* < 0.001 and ^∗∗^*p* < 0.05.

In order to evaluate the therapeutic effect of miRNA-101-3p, agomir-101-3p was injected in situ in the rats' coccygeal vertebrae and the radiographic and histological analysis after 28 days from injection was conducted to evaluate the degree of disc degeneration. As shown in Figure 5(a), the intervertebral disc height of the IDD group was significantly decreased compared to that of the NC group, whereas the intervertebral disc height of the IDD treated with agomir-101-3p showed a certain degree of increase compared to that of the IDD group, which further indicated the miRNA-101-3p could attenuate the degeneration of disc. Further statistical analysis of disc height index results also showed that agomir-101-3p mimic treatment could better maintain intervertebral height, suggesting mild degeneration (*p* < 0.001).

As for the pathological histological staining (Figure 5(b)), HE staining showed that the basic structure of NP disappeared in the IDD group, and the surrounding area has no clear boundary between the annulus fibrosus and NP; NP cells have been degenerated or replaced by cells of fibroblast-like phenotype. The degeneration degree of the agomir-101-3p treatment group was relatively mild even though the number and intact structure of NP cells were still decreased and broken compared to the NC group. And similar results were obtained by Safranin-O-fast green staining. In the IDD group, intervertebral discs were found severely degenerated with a large number of green staining fibrous tissues. In the agomir-101-3p treatment group, increased orange staining ECMs were found in the NP, with a small amount of degenerative fibrous tissue. Corresponding to the pathological micrographs mentioned above, the histologic grading scores of the agomir-101-3p treatment group were significantly lower than those of the IDD group (*p* < 0.05), suggesting the inhibition effects of miRNA-101-3p on the progression of IDD.

## 4. Discussion

As housekeeping and regulatory noncoding RNAs, microRNA has been reported to be closely related to the prognosis and progression of many human diseases. It is an important regulatory molecule in cell physiological and pathological processes, playing an important role in cell proliferation, apoptosis, differentiation, and carcinogenesis [21, 22]. By binding to the 3′-UTR of the target mRNA, miRNA partially or completely inhibits the translation process and even directly silences the expression of the target gene. Therefore, miRNA has the potential to become a target of gene therapy in the future [23]. With the deepening researches for IDD mechanism, a growing number of studies have found that the differential expression of various miRNAs in normal and degenerated human intervertebral disc tissue may lead to NP cell apoptosis, degeneration of extracellular matrix, and inflammation, suggesting that miRNA is one of the indispensable regulatory molecules in the mechanism of IDD [5, 24–26].

Stanniocalcin (STC) is a kind of glycoprotein hormone which was first found from the corpuscles of stannius of teleost fishes [27]. Many studies have shown that STC plays an important role in many biological processes, such as calcium regulation, osteogenesis, oxidative stress, anti-inflammation, angiogenesis, ischemia-reperfusion injury, tumor proliferation, and drug resistance [28–34]. And recent research has reported that upregulation of stanniocalcin-1 can inhibit the development of osteoarthritis [35], but to our best knowledge, there is still no study that has reported the correlation between STC and IDD by miRNA regulation. In this research, our findings first pointed out that the miRNA-101-3p expression level was significantly decreased in the IDD tissue and upregulation of the miRNA-101-3p could alleviate the IDD process by the STC1/VEGF/MAPK pathway, providing a novel IDD pathogenetic mechanism (Figure 5(c)).

Previous studies have reported that miR-101 is involved in a variety of human physiological and pathological processes [36–41]; in order to enhance the reliability of our study, the miRNA microarrays were employed to screen the differentially expressed miRNA; thereby, the miRNA-101-3p was finally chosen. Subsequently, the downstream potential target genes of miRNA-101-3p were predicted by the miRNA databases according to the sequence complementarity algorithms. STC1 was selected to be the promising candidate at last. To verify the binding efficiency, the luciferase reporter assay analysis was performed and the results suggested that miR-101-3p could directly bind to the 3′-UTR of STC1 mRNA and effectively inhibit the STC1 expression.

Since numerous researches had illustrated that the intervertebral disc degeneration and inflammation mechanism was related to disc angiogenesis [42–44], one of the conventional angiogenic factors, VEGF, was selected to be the promising downstream molecule for further study. Then, a series of PCRs and western blots were carried out to validate each corresponding mRNA and protein expression. The results demonstrated that the STC1/VEGF expression level was elevated in the IDD tissue with a low expression level of miRNA-101-3p. However, when the expression level of miR-101-3p was upregulated by the miRNA-101-3p mimics, the STC1/VEGF/MAPK pathway expression level dropped off, with the conventional ECM degrading enzymes such as MMP3 reduced in the meantime. Moreover, the animal experiment was also conducted to further verify the effect of miR-101-3p, by injecting the agomir-101-3p into the degenerated rats' coccygeal vertebrae; the less degenerated pathological images with a relatively lower histological score were obtained compared with the IDD group. All the findings above strongly indicated that miR-101-3p was a significant molecular regulator in the pathogenetic mechanism of IDD and may also have the potential to be the target of IDD gene therapy.

Through this study, we elucidated the pivotal role of miR-101-3p in the STC1/VEGF/MAPK pathway that may be responsible for IDD. However, even if there are increasing researches that have reported various kinds of miRNAs were involved in the IDD process, it is still a huge challenge to implement it in clinical practice. Considering the biodegradability and potential off-target effects of miRNAs, a suitable vehicle with high in vitro gene transfection efficiency and negligible toxicity is necessary. In addition, it is known that microRNA can be regulated on the transcriptional level, such as DNA methylation, influenced by the endogenous factors or xenobiotics [45, 46]. In this study, the upstream regulation mechanism of the downregulation of miR-101-3p in IDD tissue was not clearly investigated. What is more, although the rat animal models used in our study are easily prepared with great reproducibility, the rat caudal discs are different from those of humans in mechanical loading, anatomy, and composition of disc [47]. Therefore, further study is needed to illustrate a more comprehensive mechanism of miR-101-3p in IDD.

In summary, the proof-of-concept study demonstrated an IDD-related miRNA, miR-101-3p, screening by the next-generation sequencing (NGS), which remarkedly inhibits disc degeneration progression through the STC1/VEGF/MAPK signaling pathway. These results provided a novel molecular mechanism involving NP cell apoptosis and a potential treatment strategy for the pathogenesis of IDD.

## Data Availability

All data during the study are available from the corresponding author by request.

## Supplementary Materials

Supplementary Materials Table S1: differentially expressed miRNAs in NP tissues from the IDD and NC groups in both first- and second-stage validation. Table S2: primers used for quantitative real-time polymerase chain reaction. Table S3: Pfirrmann classification [8]. Figure S1: (A) the different expression levels of STC1 and VEGF in the IDD and NC groups analyzed by qRT-PCR assay. ^∗∗∗^*p* < 0.01. (B) PPI (protein-protein interaction) network shows the interaction between STC1 to VEGF and MAPK.
