# Supplementary material for: The Regulatory Effect of MicroRNA-101-3p on Disc Degeneration by the STC1/VEGF/MAPK Pathway
Source: Oxid Med Cell Longev. 2021 Oct 5;2021:1073458. doi: 10.1155/2021/1073458 (PMC8510813; doi:10.1155/2021/1073458)
Supplement: Supplementary Materials — Table S1: differentially expressed miRNAs in NP tissues from the IDD and NC groups in both first- and second-stage validation. Table S2: primers used for quantitative real-time polymerase chain reaction. Table S3: Pfirrmann classification [8]. Figure S1: (A) the different expression levels of STC1 and VEGF in the IDD and NC groups analyzed by qRT-PCR assay. ∗∗∗p < 0.01. (B) PPI (protein-protein interaction) network shows the interaction between STC1 to VEGF and MAPK. [file 1073458.f1.docx]

**Table S1**

Differentially expressed miRNAs in NP tissues from IDD and NC group in both first- and second-stage validation.

| miRNAs | First stage validation | | Second stage validation | |
| --- | --- | --- | --- | --- |
|  | Fold change | P value | Fold change | P value |
| **Up-regulated** |  |  |  |  |
| **has-miR-29b-3p** | **8.9** | **0.009^**^** | 9.2 | 0.15 |
| has-miR-375-3p | 2.9 | 0.13 | – | – |
| **has-miR-141** | **7.4** | **0.007^**^** | 6.7 | 0.23 |
| has-miR-122-5p | 5.2 | 0.25 | – | – |
| has-miR-155 | 6.3 | 0.46 | – | – |
| has-miR-135a-5p | 2.4 | 0.57 | – | – |
| has-miR-137-3p | 6.5 | 0.09 | – | – |
| **has-miR-146b-5p** | **8.0** | **0.005^**^** | 10.3 | 0.42 |
| has-miR-192 | 4.1 | 0.34 | – | – |
| has-miR-190a-5p | 7.9 | 0.28 | – | – |
| has-miR-33-5p | 3.3 | 0.19 | – | – |
| **Down-regulated** |  |  |  |  |
| has-miR-152-3p | 0.27 | 0.43 | – | – |
| has-miR-136-3p | 0.31 | 0.26 | – | – |
| has-miR-541 | 0.43 | 0.17 | – | – |
| has-miR-411-5p | 0.24 | 0.08 | – | – |
| has-miR-127-3p | 0.33 | 0.51 | – | – |
| has-miR-379 | 0.18 | 0.38 | – | – |
| has-miR-199a-5p | 0.22 | 0.12 | – | – |
| **has-miR-101-3p** | **0.11** | **0.003^**^** | **0.009** | **0.002^**^** |
| has-miR-455-5p | 0.48 | 0.24 | – | – |
| has-miR-181b-5p | 0.39 | 0.35 | – | – |
| has-miR-338 | 0.41 | 0.49 | – | – |

^⁎⁎^ P < 0.01.

**Table S2**

Primers used for quantitative real-time polymerase chain reaction.

| Gene | Forward Primers | Reverse Primers |
| --- | --- | --- |
| miR-101-3p | 5ʹ-TCCGAAAGTCAATAGTGTC-3ʹ | 5ʹ-GTGCAGGGTCCGAGGT-3ʹ |
| STC1 | 5ʹ- GCAGGAAGAGTGCTACAGCAAG-3ʹ | 5ʹ- CATTCCAGCAGGCTTCGGACAA-3ʹ |
| VEGF | 5ʹ- GCACCCATGGCAGAAGG-3ʹ | 5ʹ- CTCGATTGGATGGCAGTAGCT-3ʹ |
| U6 | 5ʹ-CTCGCTTCGGCAGCACA-3ʹ | 5ʹ-AACGCTTCACGAATTTGCGT-3ʹ |

**Table S3 Pfirrmann classification ^[1]^**


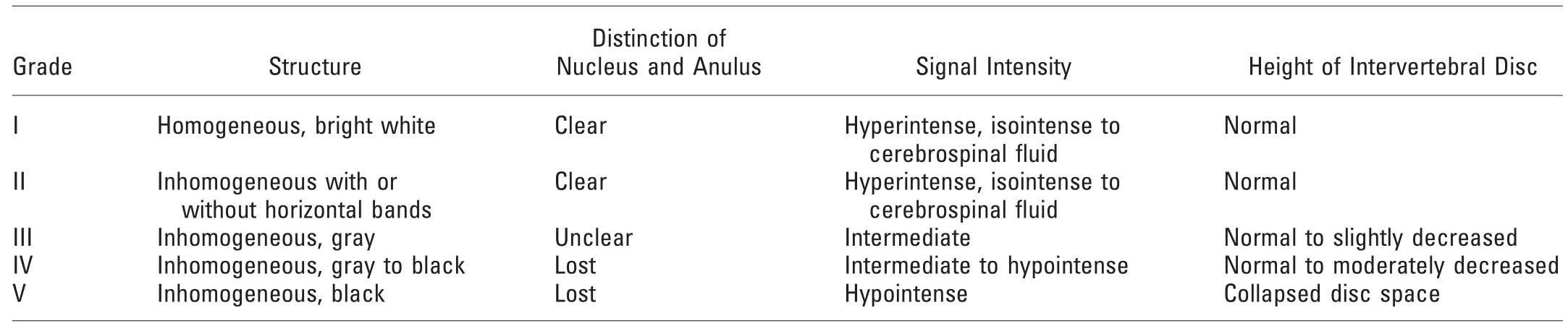


[1] Pfirrmann CW, Metzdorf A, Zanetti M, Hodler J, Boos N. Magnetic resonance classification of lumbar intervertebral disc degeneration. Spine (Phila Pa 1976). 2001;26:1873–8.

**Figure S1
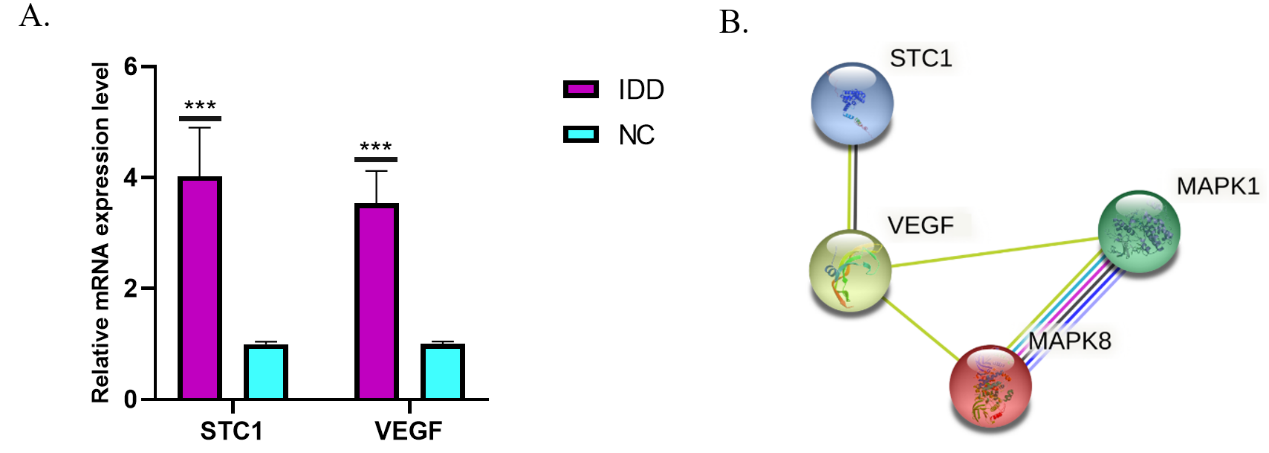
**

**Figure S1** :(A) The different expression level of STC1 and VEGF in IDD and NC groups analyzed by qRT-PCR assay. ***P < 0.01 (B) PPI (Protein-protein interaction) network shows the interaction between STC1 to VEGF and MAPK.
